# Engaging Caregivers and Providers of Children With Sickle Cell Anemia in Shared Decision Making for Hydroxyurea: Protocol for a Multicenter Randomized Controlled Trial

**DOI:** 10.2196/27650

**Published:** 2021-05-21

**Authors:** Anna M Hood, Heather Strong, Cara Nwankwo, Yolanda Johnson, James Peugh, Constance A Mara, Lisa M Shook, William B Brinkman, Francis J Real, Melissa D Klein, Rogelle Hackworth, Sherif M Badawy, Alexis A Thompson, Jean L Raphael, Amber M Yates, Kim Smith-Whitley, Allison A King, Cecelia Calhoun, Susan E Creary, Connie M Piccone, Aimee K Hildenbrand, Steven K Reader, Lynne Neumayr, Emily R Meier, Amy E Sobota, Sohail Rana, Maria Britto, Kay L Saving, Marsha Treadwell, Charles T Quinn, Russell E Ware, Lori E Crosby

**Affiliations:** 1 Developmental Neurosciences Institute of Child Health University College London London United Kingdom; 2 Behavioral Medicine and Clinical Psychology Cincinnati Children's Hospital Medical Center Cincinnati, OH United States; 3 Department of Psychology Oklahoma State University Stillwater, OK United States; 4 Department of Pediatrics University of Cincinnati College of Medicine Cincinnati, OH United States; 5 Division of Hematology Cincinnati Children's Hospital Medical Center Cincinnati, OH United States; 6 Division of General and Community Pediatrics Cincinnati Children's Hospital Medical Center University of Cincinnati College of Medicine Cincinnati, OH United States; 7 Cincinnati Children's Hospital Medical Center Partner Cincinnati, OH United States; 8 Department of Pediatrics Feinberg School of Medicine Northwestern University Chicago, IL United States; 9 Division of Hematology, Oncology and Stem Cell Transplant Ann & Robert Lurie Children's Hospital of Chicago Chicago, IL United States; 10 Center for Child Health Policy and Advocacy Baylor College of Medicine Houston, TX United States; 11 Department of Pediatrics Division of Hematology/Oncology Baylor College of Medicine Houston, TX United States; 12 Children’s Hospital of Philadelphia Philadelphia, PA United States; 13 Perelman School of Medicine Philadelphia, PA United States; 14 Program in Occupational Therapy and Pediatrics Division of Hematology and Oncology Washington University School of Medicine and St. Louis Children’s Hospital St. Louis, MO United States; 15 Division of Pediatric Hematology/Oncology Washington University School of Medicine St. Louis, MO United States; 16 Center for Innovation in Pediatric Practice Division of Pediatric Hematology/Oncology/BMT Nationwide Children's Hospital Columbus, OH United States; 17 University Hospitals Rainbow Babies and Children's Hospital Cleveland, OH United States; 18 Center for Healthcare Delivery Science Nemours Children’s Health System Wilmington, DE United States; 19 Division of Behavioral Health Nemours/ Alfred I duPont Hospital for Children Wilmington, DE United States; 20 Department of Hematology/Oncology Children's Hospital Oakland Oakland, CA United States; 21 AbbVie North Chicago, IL United States; 22 University of California San Francisco San Francisco, CA United States; 23 Pediatrics, Pediatric Hematology/Oncology Indiana Hemophilia and Thrombosis Center Indianapolis, IN United States; 24 Pediatric Hematology and Oncology Boston Medical Center Boston, MA United States; 25 Pediatrics and Child Health College of Medicine Howard University Washington, DC United States; 26 Adolescent and Transition Medicine Cincinnati Children’s Hospital Medical Center Cincinnati, OH United States; 27 James M. Anderson Center for Health Systems Excellence Cincinnati Children’s Hospital Medical Center Cincinnati, OH United States; 28 Department of Pediatrics University of Illinois College of Medicine Peoria, IL United States; 29 Department of Hematology/Oncology University of California San Francisco Benioff Children’s Hospital Oakland, CA United States; 30 Cancer and Blood Diseases Institute Division of Hematology Cincinnati Children’s Hospital Medical Center Cincinnati, OH United States

**Keywords:** dissemination, decisional uncertainty, quality of care, child health, NHLBI guidelines

## Abstract

**Background:**

Sickle cell anemia (SCA) is a genetic blood disorder that puts children at a risk of serious medical complications, early morbidity and mortality, and high health care utilization. Until recently, hydroxyurea was the only disease-modifying treatment for this life-threatening disease and has remained the only option for children younger than 5 years. Evidence-based guidelines recommend using a shared decision-making (SDM) approach for offering hydroxyurea to children with SCA (HbSS or HbS/β0 thalassemia) aged as early as 9 months. However, the uptake remains suboptimal, likely because caregivers lack information about hydroxyurea and have concerns about its safety and potential long-term side effects. Moreover, clinicians do not routinely receive training or tools, especially those that provide medical evidence and consider caregivers’ preferences and values, to facilitate a shared discussion with caregivers.

**Objective:**

The aim of this study is to understand how best to help parents of young children with sickle cell disease and their clinicians have a shared discussion about hydroxyurea (one that considers medical evidence and parent values and preferences).

**Methods:**

We designed our study to compare the effectiveness of two methods for disseminating hydroxyurea guidelines to facilitate SDM: a clinician pocket guide (ie, usual care) and a clinician hydroxyurea SDM toolkit (H-SDM toolkit). Our primary outcomes are caregiver reports of decisional uncertainty and knowledge of hydroxyurea. The study also assesses the number of children (aged 0-5 years) who were offered and prescribed hydroxyurea and the resultant health outcomes.

**Results:**

The Ethics Committee of the Cincinnati Children’s Hospital Medical Center approved this study in November 2017. As of February 2021, we have enrolled 120 caregiver participants.

**Conclusions:**

The long-term objective of this study is to improve the quality of care for children with SCA. Using multicomponent dissemination methods developed in partnership with key stakeholders and designed to address barriers to high-quality care, caregivers of patients with SCA can make informed and shared decisions about their health.

**Trial Registration:**

ClinicalTrials.gov NCT03442114; https://clinicaltrials.gov/ct2/show/NCT03442114

**International Registered Report Identifier (IRRID):**

DERR1-10.2196/27650

## Introduction

### Background

Sickle cell anemia (SCA) is a genetic blood disorder that affects approximately 100,000 individuals in the United States [1]. It is the most common disorder identified by newborn screening, with approximately 1 in 2000 babies born with SCA in the United States each year [2]. SCA is a chronic disease that is associated with significant morbidity and early mortality [3]. Until 2017, hydroxyurea was the only approved disease-modifying treatment for individuals with SCA, and it has remained the only treatment option for children younger than 5 years [4]. Hydroxyurea has many beneficial effects, including reduced pain and acute chest syndrome episodes, reduced hospital admissions, and less need for blood transfusions, among patients with SCA [5,6]. In 2014, the National Heart, Lung, and Blood Institute (NHLBI) published guidelines that recommended clinicians offer hydroxyurea to children with SCA (HbSS and HbS/β0 thalassemia), beginning as early as 9 months of age [7]. Previously, medical providers only offered hydroxyurea to children with SCA with persistent pain or other severe SCA-related complications. Despite these guidelines, hydroxyurea uptake remains low in young children with SCA [8]. Barriers to taking hydroxyurea include a lack of caregiver knowledge about hydroxyurea, providers’ hesitancy to prescribe hydroxyurea, and concerns about poor adherence to the treatment [9].

NHLBI guidelines encourage using shared decision making (SDM) when providers offer hydroxyurea, which involves a collaborative process wherein clinicians, patients, and families work together to reach a mutual agreement about the course of treatment [7,10]. However, the only widely distributed tool to assist providers in implementing the NHLBI guidelines for hydroxyurea is a clinician pocket guide developed by the American Society of Hematology (ASH) [8]. The pocket guide was a critical first step, but it *only* targeted clinician motivation. Pocket guides do not provide training to build clinician self-efficacy in prescribing medications, feedback to reinforce behavior change, or decision support tools to help clinicians engage and support caregivers in SDM.

### Objectives

We developed the hydroxyurea SDM toolkit (H-SDM toolkit), which is a caregiver-centered, technology-enhanced decision support tool. We designed the H-SDM toolkit to strengthen SDM, reduce caregiver uncertainty, allay potential fears, increase the offering of hydroxyurea, and ultimately improve hydroxyurea uptake [10,11]. In this paper, we present a protocol for a multisite randomized controlled trial (RCT; ENGAGE HU). The objective of the ENGAGE HU trial is to determine whether the use of the H-SDM toolkit is more effective than the ASH clinician pocket guide (ie, usual care) as a dissemination method. This study aims to improve the quality of the available evidence so that caregivers and providers have the appropriate tools to make an informed decision about hydroxyurea.

## Methods

### Framework

The Reach, Effectiveness, Adoption, Implementation, and Maintenance (RE-AIM) model provides a framework for evaluating dissemination methods that can improve the sustainable adoption and implementation of effective, generalizable, and evidence-based interventions by attending to 5 factors: (1) *Reach*: does the intervention reach the intended population?; (2) *Efficacy or effectiveness*: does the intervention impact the essential outcomes?; (3) *Adoption*: is the intervention supported by staff, settings, or institutions?; (4) *Implementation*: is the intervention delivered consistently?; and (5) *Maintenance*: what is in place to ensure that the intervention continues over time? [12]. We developed our study plan using RE-AIM because this framework improves the quality, speed, and impact of dissemination methods [13].

### Specific Aims

**Figure 1:**
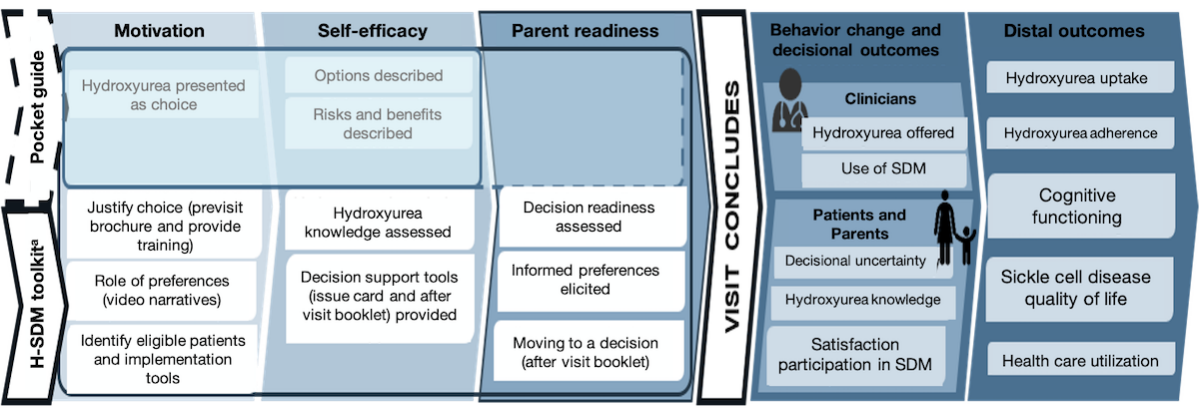
Engaging parents of children with sickle cell disease and their providers in shared decision making for hydroxyurea (ENGAGE HU) conceptual model. H-SDM: hydroxyurea shared decision making; SDM: shared decision making.

The H-SDM toolkit engages both caregivers and clinicians and targets (1) clinician motivation and self-efficacy and (2) caregiver readiness. Therefore, we propose that it will lead to change in caregiver’s and clinician’s behavior, which will increase SDM about hydroxyurea between caregivers and clinicians as well as improve decisional outcomes (ie, decisional uncertainty, hydroxyurea knowledge, and satisfaction with the decision-making process; Figure 1) [10]. If caregivers of children with SCA feel more confident and knowledgeable about hydroxyurea and more involved in the decision-making process, then they may be more likely to initiate hydroxyurea and subsequently ensure that their child adheres to the medication. Thus, the primary aims of the ENGAGE HU study are as follows:

- *Hypothesis 1:* The H-SDM toolkit dissemination method will result in greater perceptions of SDM and less uncertainty among caregivers of children with SCA than the ASH clinician pocket guide (ie, usual care).
- *Hypothesis 2:* The H-SDM toolkit dissemination method will result in greater improvements in caregiver knowledge about hydroxyurea, more children being offered and receiving hydroxyurea, and better health outcomes than the ASH clinician pocket guide (usual care).

### Overview of Study Design

**Figure 2:**
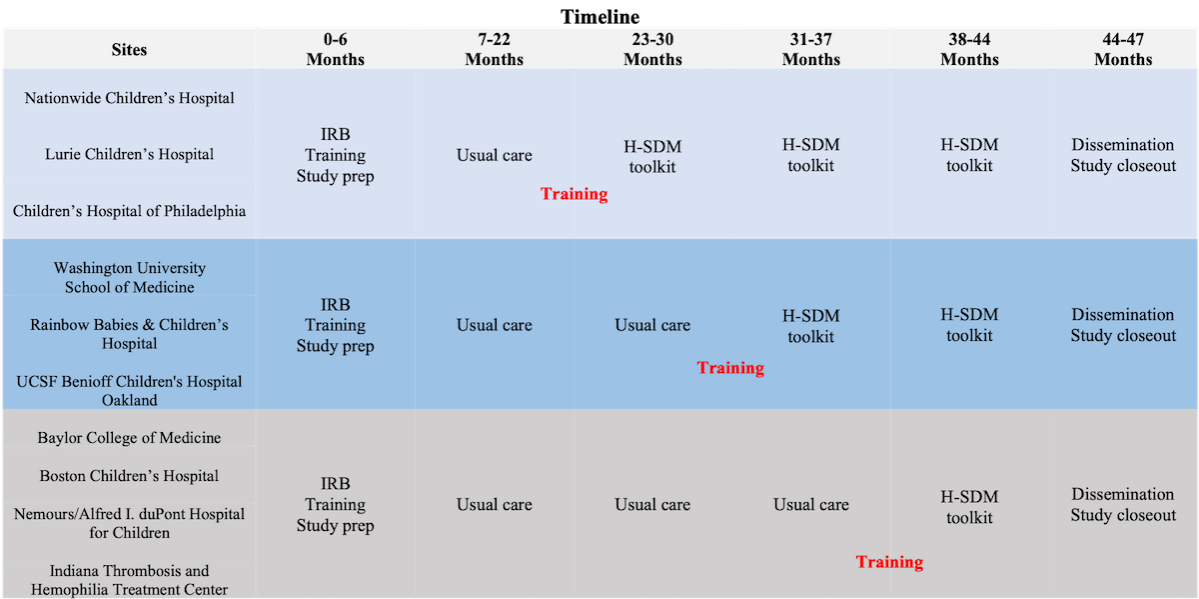
Study timeline. H-SDM: hydroxyurea shared decision making; IRB: institutional review board.

ENGAGE HU is an adapted stepped-wedge, stratified, multicenter RCT [14] with the H-SDM toolkit as the intervention and the ASH clinician pocket guide as the active comparator. We selected sites in the United States with sickle cell clinics that work with patient populations from urban, suburban, and rural communities. Each clinic begins to enroll patients using the ASH clinician pocket guide as the dissemination method. Each site then *crosses over* to using the H-SDM toolkit (Figure 2). Clinician training for the H-SDM toolkit begins during the last month of each usual care period. A stepped-wedge design with sequential assignment was chosen to ensure that all sites received the intervention and could *step in* at different timepoints. All sites will complete the usual care condition first, as clinicians would not be able to unlearn skills gained from the H-SDM toolkit. Each site enrolls approximately 4-5 participants per period. Enrollment ends 44 months after the study initiation (Figure 2).

### Randomization

We chose an adapted stepped-wedge randomized trial (clinic is the unit of randomization) with sites not previously exposed to the H-SDM toolkit. This design maximizes our confidence that differences in outcomes between the groups occur due to the dissemination method and not baseline discrepancies among participant groups, confounding, or chance. We randomize at the site level to reduce the risk of contamination and because the NHLBI guidelines require clinics to make system-level changes to deliver high-quality care. The order in which the sites crossover is random (Figure 2). The randomization minimizes differences among large (>501 patients), medium (270-500 patients), and small (<270 patients) sites.

### Patient Involvement

Patients with SCA and their caregivers provided input at several stages of the trial, including development, design, feasibility, and trial conduct. They also assisted with the toolkit components, choice of outcome measures, and recruitment methods. They helped us carefully assess the trial burden on caregivers of patients with SCA. Stakeholders will be coauthors of peer-reviewed publications, and the study results in a format suitable for a nonspecialist audience will be sent to the patients and caregivers.

### Eligibility and Consent

Eligibility criteria for the caregiver participants in the ENGAGE HU study include the following: their child has a diagnosis of SCA and receives care at the recruitment site; their child is aged 0-5 years; their child is eligible for hydroxyurea according to NHLBI guidelines (HbSS and HbS/β0 thalassemia); they can participate in both study visits; and they can read, understand, and speak English fluently. Exclusion criteria include their child has an active hydroxyurea prescription filled in the past 3 months; they have previously made a decision about whether to initiate hydroxyurea (after November 2019, we changed this criterion to include whether the research team had approached the caregiver reinitiation of hydroxyurea within the past 3 months); any diagnoses or conditions that, in the opinion of the site investigator or hematologist, would prevent the patient from being a suitable candidate for the study; and their child is a sibling of a participant actively or previously enrolled in the study.

The Cincinnati Children’s Hospital Medical Center (CCHMC) is the co-ordinating center (CC) for this multisite trial (Multimedia Appendix 1 [14-39]). The CCHMC oversees the study conduct, regulatory and institutional review board (IRB) administration, and compliance. The protocol for ENGAGE HU includes an IRB-approved waiver of the documentation of consent for clinicians participating in the trial. The trial also has an IRB-approved waiver of consent that permits clinical sites to generate a list of eligible patients. Caregivers participate in the informed consent process and are required to provide written or electronic consent.

### Recruitment and Retention

The ENGAGE HU trial consecutively enrolls caregivers of patients with SCA across the United States. Caregivers are identified as potentially eligible for hydroxyurea by provider referral or electronic health record review. A member of the site research team approaches the child’s hematology provider to obtain approval before contacting the potential caregiver participant. Caregivers of patients deemed eligible will receive an invitation via regular mail or phone or may be approached during a clinic visit, with research coordinators at each site screening participants and completing the informed consent process.

Evidence-based strategies for optimizing participation and retention include scheduling visits at times convenient for the family, reminder phone calls, allowing participants to complete questionnaires on the internet, scheduling phone or video problem-solving sessions at a convenient time, and *check-in* calls during the COVID-19 pandemic. In addition, the importance of the follow-up visit is reviewed with each family at baseline to engage them as partners in the research process. We also seek to aid sites in developing solutions to reach nonresponders during weekly study meetings. To improve retention, we collect multiple forms of contact information from multiple contacts (eg, family members and friends) to stay in close contact with families. In addition, we use a graduated incentive system for visits to reduce attrition. A Stakeholder Advisory Council, whose members include the caregivers of patients with SCA, reviews and provides ongoing feedback on the recruitment and retention plan.

### Hydroxyurea Dissemination Methods

#### H-SDM Toolkit

Guided by the social cognitive theory, and considering preidentified barriers [40], the team designed the H-SDM toolkit to increase the likelihood that caregivers and health care providers would engage with one another to make a joint decision about hydroxyurea. The social cognitive theory posits that behavior change occurs when an individual is motivated, feels confident in his or her ability to perform the new behavior (self-efficacy), and observes that the behavior is successfully performed and reinforced by others (observational learning and reinforcement). The development of the H-SDM toolkit is described in detail elsewhere [11]. Briefly, our research team collaborated with clinicians, educators, community-based organizations, and patients with SCA and their caregivers to identify barriers related to decision making regarding hydroxyurea. We designed the H-SDM toolkit with core and optional components tailored to individuals’ needs while also being broadly applicable (Table 1).

**Figure 3:**
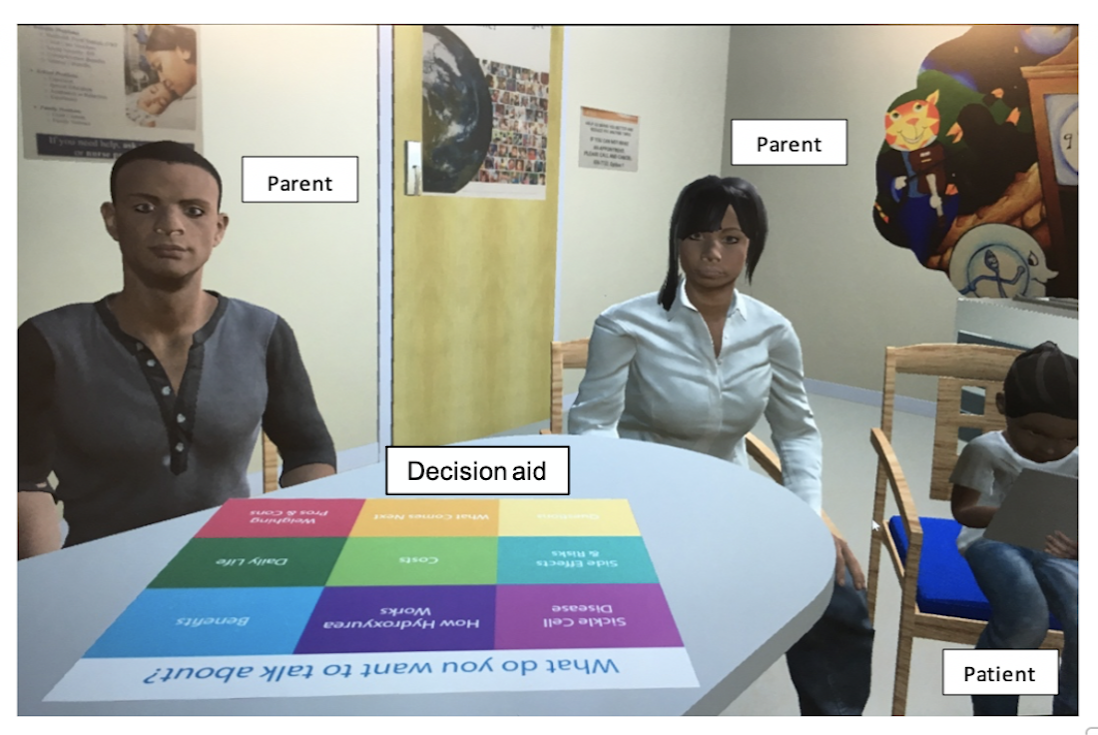
The clinical environment viewed through the virtual reality headset.

Clinicians participate in guided practice using the visit decision aids in an immersive virtual reality environment [41] (Figure 3). The virtual reality simulation was adapted from a general pediatric practice protocol to train clinicians to discuss the influenza vaccine with racial and ethnic minority families [42].

**Table 1:** Hydroxyurea shared decision-making toolkit components.

Materials	Component	Process	Core	Optional
Virtual reality simulation	Guided practice	Clinicians receive virtual reality training to increase their self-efficacy in describing hydroxyurea risks, benefits, and other treatments; eliciting caregiver preferences; assessing decision readiness; and moving caregivers toward a decision	✓^a^	N/A^b^
Previsit brochure	Decision aid	To increase caregiver motivation to make a decision by providing information about hydroxyurea as a treatment option	N/A	✓
In-visit issue card	Decision aid	To increase caregiver self-efficacy by providing them with the information needed to evaluate the benefits and risks of hydroxyurea and other treatment options	✓	N/A
After-visit booklet	Decision aid	Includes links to reputable resources; caregivers can take notes and take this resource home to share with other caregivers involved in decision making	✓	N/A
Parent video narratives	Decision aid	Four videos (3 mothers and 1 father)—caregivers telling their story about how they made a decision about hydroxyurea	N/A	✓
Previsit planning template	Identifying eligible patients	Secure SharePoint site with electronic health record templates (eg, EPIC builds)	N/A	✓
Care gap report template	Identifying eligible patients	Secure SharePoint site with tools to identify eligible patients who were missed or not approached	N/A	✓
Checklist template	Identifying eligible patients	Secure SharePoint site with a SCA^c^ data collection form for tracking whether hydroxyurea was offered and prescribed	N/A	✓
Process map template	Implementation	Sites are provided with quality improvement tools that help integrate guidelines into their care delivery system. Process maps visually describe the flow of work or ideas	✓	N/A
Failure mode effect analysis template	Implementation	Failure mode effect analysis templates assist teams in determining how their clinic process needs to change to incorporate the NHLBI^d^ guidelines into routine care	N/A	✓
Plan-do-study-act template	Implementation	Plan-do-study-act templates assist teams in determining how their clinic process needs to change to incorporate the NHLBI guidelines into routine care	N/A	✓
Key driver diagram template	Implementation	Site teams complete key driver diagrams, which are a visual display of a team’s theory of what “drives” or contributes to the study aims	N/A	✓
Implementation planning tool	Implementation	Teams are invited to weekly calls and booster sessions to review best practices in the implementation, particularly for the clinical decision support	✓	N/A
Run chart template 1	Monitoring	For use in shared decision making	✓	N/A
Run chart template 2	Monitoring	To track eligible patients who have been offered hydroxyurea	✓	N/A
Run chart template 3	Monitoring	To track hydroxyurea prescriptions	N/A	✓
Hydroxyurea navigator monitoring tool (dose, labs, and adherence)	Monitoring	Table of dates and laboratory values with a way for clinicians to indicate if a value is in range or moving in the right direction	N/A	✓

^a^Check marks indicate whether a component of the toolkit is core or optional.

^b^N/A: not applicable.

^c^SCA: sickle cell anemia.

^d^NHLBI: The National Heart, Lung, and Blood Institute.

#### Usual Care

The ASH clinician pocket guide contains information that may motivate clinicians to use an SDM approach (eg, the NHLBI guidelines). The guide also includes information that may increase clinician confidence in describing hydroxyurea risks, benefits, and other treatments to caregivers. All sites receive printed copies of the guide, a link to download copies, and a link to the app to distribute to their clinicians. Site clinicians also view a live or recorded didactic presentation that reviews the NHLBI guidelines for hydroxyurea. Sites then develop or update their site-specific care guidelines for hydroxyurea and create a plan for implementation.

### Procedures

Once the research team obtains consent from interested caregivers, they can complete baseline assessments at that time or a more convenient date. If caregivers prefer or cannot complete measures in person, then they can complete them on internet using Research Electronic Data Capture (REDCap) [43]. If a caregiver does not complete baseline assessments within 30 days of consent, then the caregiver may be rescreened, reconsented, and asked to complete baseline assessments one more time. At baseline, caregiver participants discuss initiating hydroxyurea with providers, and measures may be completed over 1-2 visits with 3 baseline visit types: (1) Full baseline: caregiver completes all baseline measures during one visit, (2) Baseline part 1: caregiver participants complete baseline measures not dependent upon the hydroxyurea discussion, and (3) Baseline part 2: caregiver participants complete baseline measures relevant to hydroxyurea discussion (Table 1).

**Figure 4:**
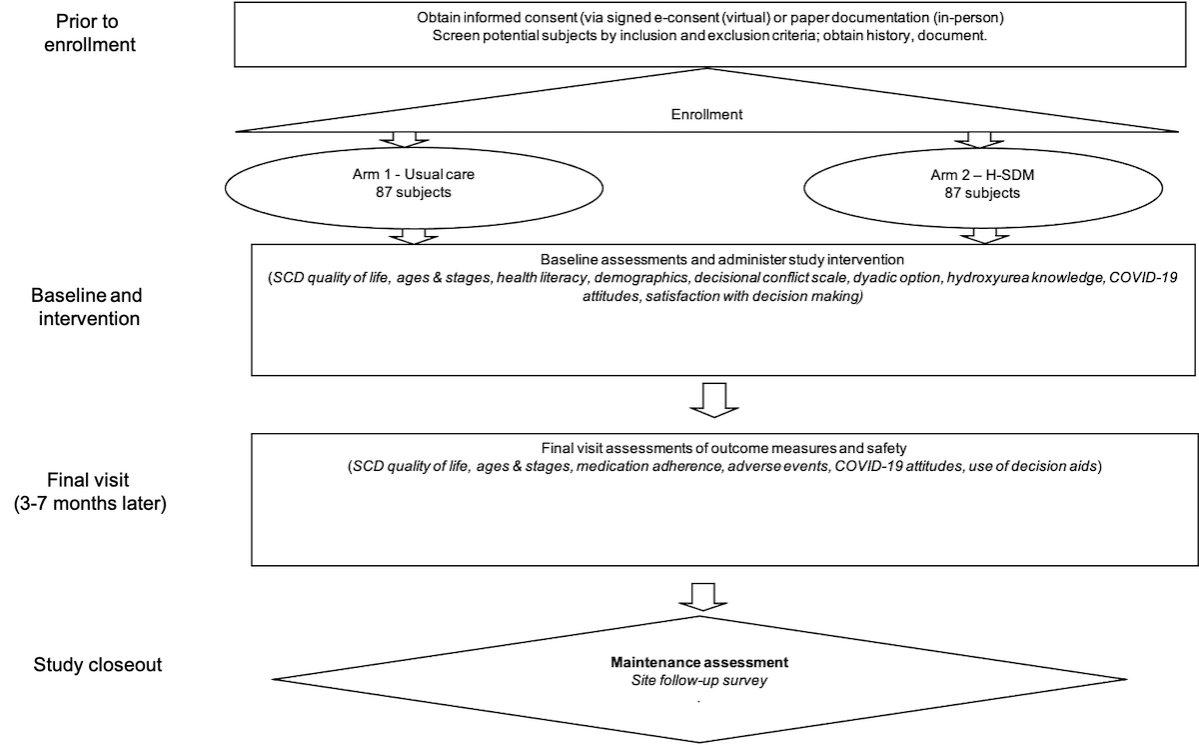
Study flowchart. H-SDM: hydroxyurea shared decision making; SCD: Sickle Cell Disease.

The follow-up visit occurs between 3 and 7 months after baseline, and caregiver participants can complete measures during a clinic visit or on the internet. After both the baseline and follow-up visits, clinicians or research staff document whether they offered or prescribed hydroxyurea, whether they used Usual Care or H-SDM toolkit, patient’s health care utilization, and hematology lab values (Figure 4). Study sites complete a follow-up survey to assess whether and how their site is continuing to implement guidelines and whether they are offering hydroxyurea 3 months after recruitment ends. Caregiver participants are compensated US $40 for completion of baseline measures, US $20 for partial completion, US $20 for hydroxyurea discussion, and US $40 for completing all follow-up measures. We also compensate caregiver participants US $5 each time they refilled hydroxyurea between baseline and follow-up time points, verified by electronic medical record review.

### Primary Outcomes

The decisional conflict scale (DCS) is a 16-item measure that assesses decisional uncertainty [15]. Items are rated on a 4-point Likert scale of 0=*strongly agree* to 4=*strongly disagree*. To calculate the total score, the 16 items are summed, divided by 16, and multiplied by 25. The scores range from 0 to 100. The dyadic OPTION (observing patient involvement) scale is a measure of the caregiver perception of clinician behaviors involved in SDM [16]. Parents respond to the following item: “My doctor and I made the decision together.” The scores range from 0 to 100 (Table 2).

**Table 2:** Primary and secondary outcome measures and covariates and fidelity assessments completed at baseline and follow-up visits in the engaging caregivers and providers of children with sickle cell anemia in shared decision making for hydroxyurea trial.

Construct			Measure		Brief description		Baseline		Follow-up
**Primary outcomes**									
	Caregiver-reported decisional uncertainty	Decisional conflict scale [15]–effectiveness		Measures uncertainty experienced when feeling uninformed about options, unclear about personal values, or unsupported in making a choice		✓^a^		N/A^b^	
	Caregiver-reported perception of shared decision making	Dyadic OPTION [16]–effectiveness		Describes clinician behaviors to involve a patient or caregiver in decision making		✓		N/A	
**Secondary outcomes**									
	Caregiver-reported satisfaction with decision making	6-item survey [17,44]–effectiveness		Three items adapted from the empirical research related to procedural justice and 3 items assessing the influence of faith on decision making		✓		✓	
	Caregiver-reported hydroxyurea knowledge	8-item survey–effectiveness		Hydroxyurea knowledge survey (8 items): developed based on the existing literature, the Ottawa Knowledge User Manual, and it was used by caregiver and clinician stakeholders in our pilot work		✓		✓	
	Caregiver report of SCD^c^-specific quality of life and pain	PedsQL^d^ SCD module [18]–effectiveness		Measures several domains of health-related quality of life including pain impact, fatigue, pain management, emotions, communication, and treatment adherence		✓		✓	
	Caregiver report of neurocognitive functioning	Ages and stages questionnaire [45]–effectiveness		Reliable, accurate developmental and social emotional screener for children aged between 2 and 60 months		✓		✓	
	Caregiver report of hydroxyurea adherence	Medical adherence measure subscale [46]–effectiveness		A 9-item survey that measures adherence problems and the extent of nonadherence in pediatric populations		N/A		✓	
	Caregiver report of continued use of decision aids	H-SDM^e^ follow-up survey		For the H-SDM condition, caregiver report of continued use of decision aids: previsit brochure, postvisit booklet, and narrative videos, including sharing information with others		N/A		✓	
	Hydroxyurea uptake	Active hydroxyurea prescription–effectiveness		One item reported by the research coordinator. They report whether patients enrolled in the study have an active prescription for hydroxyurea using the EMR^f^ (prescription in the last 6 months)		✓		✓	
	Hydroxyurea adherence	Lab values and pharmacy refill records–effectiveness		Labs reported by the research coordinator based on the EMR (past 12 months): HbF^g^ level, which increases when taking hydroxyurea as prescribed, ANC^h^, which decreases when taking hydroxyurea as prescribed, and MCV^i^, which increases when taking hydroxyurea as prescribed		✓		✓	
	Hydroxyurea offered	1 item reported by research coordinator–reach		1 of 3 responses completed by the research coordinator based on a review of EMR data: hydroxyurea was not offered, offered, or previously prescribed. If not offered, coordinators choose a reason why (ie, not eligible because the patient is on transfusions)		✓		✓	
	Health care utilization	Hospitalizations, emergency room visits, and ill visits–effectiveness		EMR data on the number of hospitalizations, ill visits, and emergency room visits in the 6 months before enrollment (if possible, some participants may be 9 months of age) and the 6 months after enrollment		✓		✓	
**Covariates**									
	Demographics	Demographics survey		10-item survey assessing family demographics including patient and caregiver age, gender, race and ethnicity, socioeconomic status, insurance (public vs private), and caregiver highest level of education completed		✓		N/A	
	Health literacy	Newest vital sign [19]		Newest vital sign (3 min): tests literacy skills for both numbers and words		✓		N/A	
	Caregiver report of the effects of COVID-19	CEFIS^j^ [47]		The CEFIS was designed to be used in ongoing and new studies where COVID-19 may impact study outcomes. It conceptualizes the exposure to potentially traumatic aspects of COVID-19 and assesses the pandemic's impact on the family		✓		✓	
	Caregiver report of telemedicine use	COVID-19 and telemedicine use survey [48]		Items from the telemedicine usability questionnaire assess the impact of telemedicine on care		✓		✓	
**Fidelity**									
	Caregiver-reported fidelity	H-SDM toolkit parent checklist		Checklist to assess the toolkit components that were used during the hydroxyurea discussion with the clinician		✓		N/A	
	Research coordinator observation of decision making	Observer OPTION scale [20]–implementation		Observer quantifies clinician behaviors to involve a caregiver in decision making. A total score is calculated, ranging from 0 (no involvement) to 100 (exemplary involvement)		✓		N/A	
	Intervention fidelity	Intervention fidelity checklist–implementation		Checklist to assess if the clinician used the intervention materials		✓		N/A	
	Study site report of continued use of intervention	Follow-up survey–maintenance		Survey to assess the continued implementation of the sites' guidelines and clinical characteristics to understand barriers and facilitators to maintain implementation		N/A		1-3 months after enrollment ends	

^a^The check mark indicates that participants completed the measure either at baseline, follow-up, or both.

^b^N/A: not applicable.

^c^SCD: sickle cell disease.

^d^PedsQL: Pediatric Quality of Life.

^e^H-SDM: hydroxyurea shared decision making.

^f^EMR: emergency medical record.

^g^HbF: fetal hemoglobin.

^h^ANC: absolute neutrophil count.

^i^MCV: mean corpuscular volume.

^j^CEFIS: COVID-19 Exposure and Family Impact Survey.

### Secondary Outcomes

A 6-item survey assesses satisfaction with decision making [17,44], and an 8-item survey assesses hydroxyurea knowledge. If the Cronbach alpha for items on these scales is acceptable (≥.70), then we will sum the ratings to obtain a total score; otherwise, we will analyze items separately. The medical adherence measure assesses problems associated with hydroxyurea adherence [46]. Nonadherence and late adherence are calculated as a continuous variable (0%-100%). The Pediatric Quality of Life Inventory Sickle Cell Module [18] is a 43-item scale with 9 dimensions that assesses health-related quality of life in patients with SCA. Caregivers rated how much of a problem an issue had been for a child on a 5-point scale of *Never* to *Almost Always*. Responses are reverse scored and linearly transformed to a 0-100 scale. Total scores are the sum of the items divided by the number of items answered. The Ages and Stages Questionnaire [45] is a set of questionnaires in which caregivers’ complete questions appropriate for their child’s developmental stage. Each developmental area is scored on a 3-point scale of 0=*not yet*, 5=*sometimes*, and 10=*yes* and then totaled and compared with area cut-off scores. The H-SDM toolkit follow-up survey measures caregivers’ continued use of decision aids (Table 2).

### Covariates

Covariate analyses will include data from the caregiver participant completed Demographics Survey and the Newest Vital Sign Survey [19]. The Newest Vital Sign Survey contains 10 items that assess health literacy skills using a mix of free response, yes or no, and Likert scale questions such as “how confident are you filling out medical forms by yourself?” The research team reviews the electronic health records to collect data on hydroxyurea (ie, if offered, if there is active prescription, adherence) based on lab values in the past 12 months, pharmacy refill records, and health care utilization.

To understand how the COVID-19 pandemic may impact trial study outcomes (eg, differential levels of distress), we added 2 additional measures in May 2020. The caregiver participants complete the COVID-19 Exposure and Family Impact Survey (CEFIS) [47]. The CEFIS contains following subscales: part 1 (exposure) comprises 25 items (yes or no responses) and part 2 (impact) comprises 12 items with 10 items using a 4-point scale rating impact on caregiver participant’s and family’s life and 2 items that use a 10-point distress scale. Part 3 is an open-ended question, so that participants could expand upon their experiences. Higher scores denote a more negative impact or exposure. The caregiver participants also complete the COVID-19 and telemedicine use survey [48], which contains 24 items rated on a 7-point Likert scale of 1=*completely disagree* to 7=*completely agree*, with statements such as “telehealth improves my access to health care services.”

### Fidelity Assessments

The site study coordinator completes the intervention fidelity checklist and assesses whether the clinician used the treatment materials outlined in the protocol. The number of fidelity assessments that sites complete is determined based on site-specific expectations for enrollment. We developed the parent checklist specifically for this study to assess the H-SDM toolkit components used during the hydroxyurea discussion with the clinician. To ensure reliability, 2 research coordinators from the CC site independently code the recorded visits using the Observer OPTION scale [20]. Clinicians must obtain a score of 80 or higher. If a score of less than 80 is received, then it indicates that the clinician or site needs additional training (1-hour video conference call). Encounters will continue to be reviewed for fidelity to ensure that the clinician or site is implementing 80% of the required toolkit components. Finally, a follow-up survey 1-3 months after enrollment ends assesses whether sites have continued implementing the H-SDM toolkit guidelines.

### Data Analyses

Data quality will be maintained through double data entry from site research team members and data quality checks from the coordinating site coordinator that assesses the conformance, completeness, and plausibility of electronic REDCap data. The coordinating site manager will also monitor data quality through random inspections. Any reliability issues were addressed with additional training. Discrepancies will be resolved by checking source data and, if necessary, by returning to patient charts to correct any inaccuracies.

All analyses will be conducted using Stata version 16 [49]. Before conducting analyses, the primary and secondary outcome measures’ psychometric properties will be assessed (eg, the measures’ dimensionality). The characteristics of the sites (eg, number of clinicians) and participants (eg, health literacy) will be summarized using descriptive statistics. Additional health care utilization variables will be analyzed as count variables and examined in the exploratory analyses.

#### Primary Outcomes Analyses

To examine differences between usual care and H-SDM toolkit groups on the DCS scale assessed at a single time point during the intervention session, a linear mixed-effects regression model with a robust variance estimator and maximum likelihood estimation will be used, with observations clustered within site and alpha set to .05. To examine differences between the usual care and H-SDM toolkit groups on the OPTION scale, we will conduct a logistic mixed-effects regression model with a robust variance estimator, maximum likelihood estimation, and observations clustered within site with alpha set to .05.

#### Secondary Outcomes Analyses

Parental knowledge and child health outcomes will be analyzed using linear mixed-effect regression models, with observations clustered within the site. To account for possible type I error inflation due to a large number of secondary outcomes, we will use the Benjamini-Hochberg procedure [50] to decrease the false discovery rate, with the overall alpha set to .05. For health care utilization outcomes, generalized mixed-effect regression models will be employed, with binary outcomes estimated using logistic models and count outcomes analyzed with negative binomial models. The interaction between these demographic variables and treatment conditions will be included in the regression models. We will examine the impact of the COVID-19 pandemic on our trial outcomes by assessing the interaction between our treatment groups and scores on the CEFIS in regression models. Observations will be coded as occurring prepandemic or *during pandemic*, regardless of treatment condition assignment.

#### Subgroup Analyses

We will compare the characteristics of the following subgroups on outcome variables: (1) caregiver participants who enroll versus those who decline, (2) dropouts versus completers, (3) clinicians who adopt the H-SDM toolkit versus those who do not, (4) sites who adopt the full H-SDM toolkit versus those that adopt only the core components, (5) sites that continue to implement guidelines versus those who do not, and (6) observations collected prepandemic versus during the pandemic.

#### Process Improvement Analyses

During the H-SDM toolkit period, data are tracked on monthly run charts, which will be converted into p-charts or control charts to determine if the process of offering hydroxyurea is under control (ie, minimal variation in the data) and if there are any notable changes (ie, factors that change the process). The upper and lower control limits will be calculated as 3 sigma from the mean (ie, standard Shewhart chart method) [21]. We will consider any data point outside the control limit variation from a special cause.

#### Fidelity Analyses

We will examine the differences between sites that continue to implement the intervention components and those who discontinue the toolkit use.

#### Statistical Power

We based sample size calculations on the smallest effect sizes (Cohen *d*) reported in previous studies using the DCS (effect sizes range from *d*=0.4 to 1.2) [22], our primary outcome, and a stepped-wedge design (eg, Hussey and Hughes approach) [14,23]. We calculated power analyses using the optimal design [51] power analysis software. With our planned sample size of n=87 per group (total sample size N=174) and up to 10%-15% missing data on our primary outcome, we will have at least 80% power to detect a standardized effect size difference of *d*=0.40 between our treatment groups on our primary outcome.

#### Data and Safety Monitoring

The study oversight will be under the direction of a Data and Safety Monitoring Board (DSMB) comprising members having expertise in SDM, hematology, psychology, biostatistics as well as a parent of a young child with SCA and an adult patient living with SCA. The DSMB will assess safety and efficacy data (if applicable), along with study’s progress and data integrity. These experts will review and evaluate the accumulated data for participant safety, adverse events, and study’s conduct and progress every 6 months. The DSMB will make recommendations to the appropriate regulatory agencies (IRB and Patient-Centered Outcomes Research Institute [PCORI]) concerning the continuation, modification, or termination of the study. Given the study is low risk, there are 2 conference calls scheduled each year.

Although no adverse events are anticipated, we will ensure that each site has procedures to refer any parents who become upset to appropriate resources for follow-up. The principal investigator (PI), a clinical psychologist with experience in managing parental distress, will provide study staff and clinician training. The PI and site PI will be notified if any individual needs psychological follow-up due to study participation. The IRB at CCHMC will be notified as soon as the immediate needs of the participant are addressed. For any severe adverse events (ie, life-threatening), the study staff will inform the PI within 1 working day. All serious adverse events will be reported to the IRB within 48 hours of the event.

## Results

The ENGAGE HU trial was funded in August 2017, and the Ethics Committee of CCHMC approved the study (Approval No. 2017-6612) in November 2017. Patient recruitment started in July 2018 and will end in November 2021. The study will be completed in February 2022. As of February 2021, we have enrolled 120 caregiver participants. We expect to disseminate the findings of the trial through peer-reviewed journals in the summer of 2022.

## Discussion

### Principal Findings

The ENGAGE HU trial compares the existing ASH clinician pocket guide with the H-SDM toolkit, which is a caregiver-centered, technology-enhanced decision support toolkit to help clinicians implement SDM for hydroxyurea [11]. We designed the H-SDM toolkit components to increase the likelihood that caregivers and health care providers would make a change in their behaviors (ie, engage with one another to make a decision about hydroxyurea). The H-SDM toolkit targets the worries, fears, and uncertainty of caregivers regarding hydroxyurea initiation. Improving hydroxyurea uptake is an important issue, as it is efficacious for patients with SCA. Given the broad stakeholder input and our preliminary studies, we believe that the H-SDM toolkit dissemination method has the potential to promote SDM and enhance the quality of care provided to children with SCA [52].

### Strengths and Limitations

Several aspects of this trial will enable the rapid adoption of findings into practice. First, we developed the H-SDM toolkit with substantial clinician and caregiver input; thus, it contains components these stakeholders felt were feasible, acceptable, and essential for improving clinical care. Second, we designed the H-SDM toolkit with core and optional components so that it can be tailored to the needs of individual families and be broadly applicable across many clinical settings. Third, virtual reality simulation provides guided practice in facilitating an SDM process. It is also a low-cost clinician training intervention that will be made accessible on national SCA-focused websites shared across SCA networks. Virtual reality training can occur in person or using web-based video conferencing tools (eg, Zoom or Microsoft Teams) [41], which is especially useful in the context of the ongoing COVID-19 pandemic. Finally, sites included in this trial were selected because their patient populations mirror the larger US SCA population with respect to economics, geography, and racial and ethnic diversity. This diversity increases the applicability of our findings to nonstudy settings and increases the available study pool.

There are some limitations to this study that should be acknowledged. A critical consideration for the ENGAGE HU trial is that SCA primarily affects individuals of African and Hispanic or Latino descent. Although the ENGAGE HU trial design includes best-practice strategies for recruiting people of color in research, we may experience recruitment difficulties because potential participants are mistrustful or have child care and transportation issues [53]. Furthermore, a successful and timely completion of clinical trials in the SCA population is compounded, as it is a rare disease with a smaller pool of available participants [54]. A potential barrier to intervention fidelity is the intrinsic difficulty in changing behavior. Given our goals to alter caregiver-clinician interactions, we address this barrier through guided practice in the virtual reality environment and audio recordings of a percentage of caregiver-clinician hydroxyurea interactions. These interventions should foster fidelity. Our pilot work indicates that clinicians currently using the toolkit decision aids find them beneficial and recommend them to others [11]. Ultimately, we hope that our study findings will have a substantial impact on improving health outcomes and decreasing health care costs in pediatric SCA and other chronic conditions.

## Appendix

Multimedia Appendix 1 Peer-review report.

## Abbreviations

ASH: American Society of Hematology
CC: co-ordinating center
CCHMC: Cincinnati Children’s Hospital Medical Center
CEFIS: COVID-19 Exposure and Family Impact Survey
DCS: Decisional Conflict Scale
DSMB: Data and Safety Monitoring Board
H-SDM: hydroxyurea shared decision making
IRB: institutional review board
NHLBI: The National Heart, Lung, and Blood Institute
PCORI: Patient-Centered Outcomes Research Institute
PI: principal investigator
RCT: randomized controlled trial
RE-AIM: Reach, Effectiveness, Adoption, Implementation, and Maintenance
REDCap: Research Electronic Data Capture
SCA: sickle cell anemia
SDM: shared decision making
