# Supplementary material for: Engaging Caregivers and Providers of Children With Sickle Cell Anemia in Shared Decision Making for Hydroxyurea: Protocol for a Multicenter Randomized Controlled Trial
Source: JMIR Res Protoc. 2021 May 21;10(5):e27650. doi: 10.2196/27650 (PMC8178738; doi:10.2196/27650)
Supplement: Multimedia Appendix 1 [file resprot_v10i5e27650_app1.pdf]

From: PFA [mailto:PFA@pcori.org]
Sent: Wednesday, May 10, 2017 4:15 PM
To: Crosby, Lori <Lori.Crosby@cchmc.org>
Cc: Sponsoredprograms <Sponsoredprograms@cchmc.org>
Subject: ACTION REQUIRED: PCORI Information Request
Privileged Communication

Dear Lori Crosby:
Thank you for your interest in PCORI and for the submission of your application to the Cycle 3 2016 Communication and Dissemination Research program.

Your application was discussed during the in-person merit review panel and as part of the ongoing review process, we are requesting clarifying information related to the research plan. The questions are listed in detail below, and we request your response by 5:00 pm EST on May 24, 2017.

Request ID#: R-1609-36055

Project Title: Engaging Parents of Children with Sickle Cell Anemia and their Providers in Shared-Decision Making for Hydroxyurea

Thank you for requesting clarifying information related to the research plan. We have included our responses below in italics.

Programmatic Concerns:

1. **While figure 1 in the application provides a conceptual model, in the overall application there is a conceptual disconnect between the theories used and the focus on measuring outcomes of decision-making and shared decision-making. Please refine model and/or provide text to clarify framing the study question(s) and hypotheses, anchoring the background literature, clarifying constructs to be measured, depicting relationships to be tested, and contextualizing results.**

We propose that a dissemination method the H-SDM toolkit that is multifaceted, involves both patients/parents and clinicians, and targets cognitive factors (motivation), behavioral factors (self-efficacy) and environmental factors (awareness/patient readiness) will lead to clinician and patient/parent behavior change (decrease in decisional uncertainty, parent involvement in decision making) and – adoption use of increasing shared decision-making about hydroxyurea between parents and clinicians practice (hydroxyurea offered, use of

shared decision making, hydroxyurea offered) and improvement in decisional outcomes (decisional uncertainty, HU knowledge, satisfaction) (see Figure 1 Conceptual Model). If parents feel more confident, less uncertain, more knowledgeable about hydroxyurea, and involved in the decision to initiate hydroxyurea, then they are more likely to initiate hydroxyurea and ensure their child is adherent with the medication (see distal outcomes in Figure 1). Furthermore, if more children with SCD are offered hydroxyurea, hydroxyurea uptake should increase. Increased HU uptake means that more children with SCD would benefit from this disease-modifying treatment, and subsequently, experience less neurological impairment and better quality of life (distal outcomes – Figure 1). Ultimately, increased hydroxyurea uptake would result in fewer ill visits, emergency room visits and hospitalizations (distal outcomes – Figure 1). We have included this information on page 7 and revised the entire research plan to better reflect our conceptual model.

Figure 1. Conceptual Model

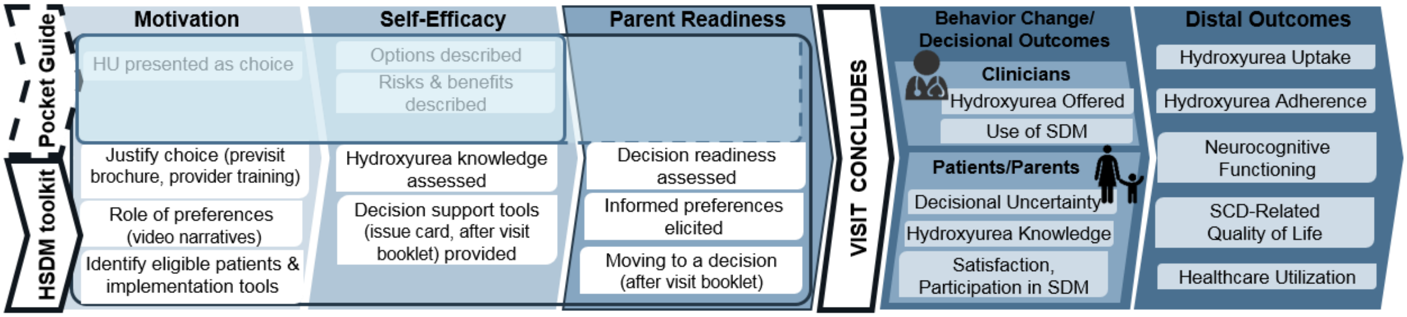

2. **It is unclear how many sites will be participating in the study. On page 9 of the application under population/settings it states "one of 9 clinics" participating. However, in the following paragraph for study design it states that there will be 4 clusters with each cluster consisting of 2 sites for a total of 8 sites. Then again on page 16, 9 sites are listed in Table 3. Participating Sites. On page 15 of the application it states data from the 9th site would be used if another site cannot meet recruitment goals. Please provide clarification on how the 8 sites for initial data collection will be selected, when data collection will begin at the 9th site, and what the criteria will be for including the 9th site if needed.**

Our initial plan was to have 8 sites participate, and to use the 9th site if another site could not meet recruitment goals. However, we now plan to have all 9 sites to

participate and have updated our study design and timeline to reflect this (see below). Inclusion of all 9 sites increases the study's power to detect changes and allows us to use a more practical intracluster correlation (ICC) (see response to #7 below). This change decreases our sample size target from 220 to 207 (225 to account for 10% attrition) and adds an additional site for recruitment. We have updated this information in the sample size calculation.

Table 1. Revised study design and timeline

	Timeline					
**Cluster**	0-6 Months	7--13 Months	14-20 Months	21-27 Months	28-33 Months	34-36 Months
**Cluster 1**<br>Sites 1, 6, 3	IRB, Training, Study Prep	Usual Care<br>Training	HSDMT	HSDMT	HSDMT	Dissemination, Study Closeout
**Cluster 2**<br>Sites 2, 7, 5	IRB, Training, Study Prep	Usual Care	Usual Care<br>Training	HSDMT	HSDMT	Dissemination, Study Closeout
**Cluster 3**<br>Sites 4, 8, 9	IRB, Training, Study Prep	Usual Care	Usual Care	Usual Care<br>Training	HSDMT	Dissemination, Study Closeout

3. **It is unclear if all of the components of the H-SDM toolkit are targeted to providers and patients. The clinician training is for the providers and the decision aids are for the parents. It is unclear who in the clinic (providers?) would be expected to use the templates for identifying eligible patients and monitoring progress as well as the implementation tools. Please clarify.**

   In our clinic, nurse care managers use the templates for identifying eligible patients, care gap report and the checklist as preplanning tools. This will be the same for all 9 SCD clinics. The implementation tools (process map, PDSA, key driver, implementation planning tool) are used by our multidisciplinary clinical team (physicians, nurses, social workers, psychologists, care coordinators, quality outcomes manager, and data analyst). Our quality outcomes manager facilitates the use of these tools but the entire team contributes. The team completes the tools during bi-weekly clinical outcomes meetings. Similarly, our multidisciplinary clinical team reviews the run charts with progress on use of shared decision-making, offering hydroxyurea, hydroxyurea prescriptions, and hydroxyurea monitoring tool during bi-weekly clinical outcomes meetings. Because each SCD clinic is different, we cannot precisely state which providers will use the implementation tools; it may be that the site Co-PI/Co-I takes the lead on these tools and completes them with input from the clinical team during existing clinical meetings or weekly study meetings. However, all sites have committed to using the Core tools to ensure that the toolkit is implemented appropriately.

4. Use of some of the RE-AIM constructs are incorrect throughout the application.

We have revised the evaluation of study using the RE-AIM framework below so that the constructs are aligned with the correct data being proposed for collection/assessment.

- **On page 11 of the application it states that "we chose an Adoption-related primary dissemination outcome: parent report of shared decision-making." Participants reporting of whether shared decision-making occurred would be considered efficacy/effectiveness of the intervention. Adoption is the proportion and representativeness of the settings (in this case clinics) and the staff who are willing to initiate the intervention(s).**

*This section now reads: "Guided by the RE-**A**IM model and with input from our clinician and advocacy stakeholders, we chose an **Efficacy/Effectiveness-related primary dissemination outcome**: **parent report of shared decision-making**."*

Table 2. Assessment Strategy				
**Construct**	**Measure**	**Brief Description/ Psychometrics (GM-4)**	**Baseline (CI-3)**	**6 mo (CI-3)**
**Primary Outcomes**				
Parent reported decisional uncertainty	Decisional Conflict Scale (DCS)[1] – **E***	Measures uncertainty experienced when feeling uninformed about options, unclear about personal values, or unsupported in making a choice. Cronbach's alpha of 0.96. (**RQ-6; PC-3**)	X	
Parent reported perception of shared decision-making	Dyadic OPTION[2] – **E***	Describes clinician behaviors to involve a patient/parent in decision-making. A total score is calculated which ranges from 0 (no involvement) to 100 (maximal involvement). Dyadic OPTION scores correlate well with OPTION scale[3] (**RQ-6; PC-3**) ; 1 item "My doctor and I made the decision together"[4] -	X	
**Secondary Outcomes**				
Parent reported Satisfaction with decision making	3 item survey – **E***	adapted from the empirical research related to the concept of procedural justice **(PC-3)**.[5] If the Cronbach's alpha for these items is acceptable (≥ .70), ratings will be summed to obtain a total score; otherwise, items will be analyzed separately. (**RQ-6**)	X	X
Parent reported Hydroxyurea knowledge	8 item survey – **E***	Hydroxyurea knowledge survey (8 items): (**PC-3**) developed based on the existing literature, the Ottawa Knowledge User Manual, parent and clinician stakeholders and used in our pilot work.[6] If the Cronbach's alpha for these items is acceptable (≥ .70), items will be summed to obtain a total score; otherwise, items will be analyzed separately. (**RQ-6**)	X	X
Hydroxyurea offered	1 item reported by research coordinator – **R***	1 of 3 responses – completed by the research coordinator based on review of EMR data: hydroxyurea was not offered, offered, or previously prescribed. If not offered, coordinators will choose a reason why (i.e. not eligible because patient is on transfusions, not eligible because patient has comorbid condition, no time to offer, clinician forgot, ill visit, or an open field to enter another reason). This will be verified for recorded encounters using the audio files. **(RQ-6)**	X	X
Hydroxyurea uptake	Active hydroxyurea prescription –**E***	1 item reported by the research coordinator. They will report whether patients enrolled in the study have an active prescription for hydroxyurea using the EMR (prescription in the last 6 months). **(RQ-6)**	X	X
Hydroxyurea adherence	Lab values & pharmacy refill records –**E***	Labs reported by the research coordinator based on the EMR: 1) fetal hemoglobin (HbF) level – fetal hemoglobin increases when taking hydroxyurea; 2) absolute neutrophil count (ANC) – this lab decreases when taking hydroxyurea. **(RQ-6)**		X
Child report of SCD-specific quality of life and pain	Peds-QL SCD Module[7] – **E***	Measures several domains of health-related quality of life including pain impact, fatigue, pain management, emotions, communication and treatment adherence; Total Score; α = .95 **(RQ-6; PC-3)**	X	X
Parent report of neurocognitive functioning	Ages & Stages Questionnaire[8] – **E***	Reliable, accurate developmental and social-emotional screener for children between birth and age 6. Cronbach's alpha ranges from .60 to .85. **(RQ-6; PC-3)**	X	X
Healthcare utilization	Hospitalizations, emergency room visits, ill visits – **E***	EMR data on the number of hospitalizations, ill visits, and emergency room visits in the 12 months prior to enrollment (if possible, some participants may be 9 months of age) and the 12 months after enrollment. **(RQ-6)**	X	X
Covariates				
Demographics	Demographics survey	10 item survey assessing family demographics including patient and parent age, gender, race and ethnicity, socioeconomic status, insurance (public vs. private), and parent highest level of education completed.	X	
Health Literacy	Newest Vital Sign[9]	Newest Vital Sign (3 minutes): tests literacy skills for both numbers and words and has been highly correlated with the REALM.[10] Cronbach's alpha = >0.76	X	
Fidelity				
Parent involvement in decision-making	Observed OPTION scale[11] – **I***	Observer quantifies clinician behaviors to involve a parent in decision-making. A total score is calculated which ranges from 0 (no involvement) to 100 (maximal involvement). OPTION scores are reliable and valid[11] (**RQ-6**). Each audiotaped clinic visit will be independently coded by two research coordinators to ensure high reliability [inter-observer agreement = 0.82 in Dr. Brinkman's recent trial.[12]	X	

Intervention fidelity	H-SDM toolkit fidelity – **I***	Checklist to assess which components of the H-SDM toolkit used and to what extent.		X
Continued use of intervention	Follow-up survey - **M**	Survey to assess continued implementation of the guidelines and clinical characteristics of the sites to understand barriers and facilitators to maintaining implementation.		X – 6 mo. after study ends

***RE-AIM Model: R = Reach; E= Effectiveness, A = Adoption; I= Implementation; M = Maintenance**

- **On page 11, Table 2. Assessment Strategy. Please correct the measures and the RE-AIM dimensions that are misaligned.**

  Please see the revised table below (also on page 14in the revised research plan):

- **On page 13 – Specific aim 2 – it states … children with an active hydroxyurea prescription (uptake – Maintenance)… This would be considered effectiveness. If maintenance is being evaluated, this would need to be described in greater detail as the number of children with an active prescription would need to be evaluated 6 months or greater post intervention period. Please clarify.**

  *Aim 2 now reads: "Specific Aim 2: Evaluate the effectiveness of the usual care dissemination method (clinician pocket guide) and the H-SDM toolkit dissemination method on: 1) parent knowledge of hydroxyurea (Effectiveness); 2) children offered hydroxyurea (Reach); 3) children with an active hydroxyurea prescription (**uptake – Effectiveness**); and 4) child health outcomes: pain, neurocognitive functioning, sickle cell related quality of life and healthcare utilization (Effectiveness). " We do not plan to measure the number of children with an active prescription 6 months or greater post intervention, which would be a maintenance measure.*

- **On page 16 of the application under D.1. Subgroup analyses #2 – characteristics of drop-outs versus completers. This is categorized as effectiveness. This is should be included in understanding reach.**
- **On page 17 - #4 – It states that characteristics of sites who adopt the full H-SDM toolkit verse the core components is considered Implementation. This is adoption. Implementation should focus on consistency of delivery of the intervention protocols.**
- **For item #5 on page 17 – which is classified as maintenance, there is no indication of when this assessment would be conducted. This is typically done 6 months or greater after the intervention has been completed. Please provided greater detail as to how maintenance will be evaluated.**

Please revise evaluation of study using this framework so that the constructs are aligned with the correct data being proposed for collection/assessment.

We have revised the evaluation plan (on page 18) as recommended below:

1. *Characteristics of parents/patients who decide to enroll versus those who decline (**Reach**)*
2. *Characteristics of drop-outs versus completers (**Reach)***
3. *Characteristics of clinicians who adopt shared decision-making versus those who do not.(**Adoption**)*
4. *Characteristics of sites who adopt the full **H-SDM toolkit** versus the core components (CRC will audit via a fidelity checklist. This will be a reported as a range [e.g. 7-9 components]) (**Adoption**)*
5. *Characteristics of settings that continue to implement guidelines versus those who do not as measured by the offering hydroxyurea measure (**Maintenance**)*

All sites will complete a follow-up survey to assess whether and how their site is continuing to implement guidelines 6 months after the study ends. The survey will ask about barriers and facilitators to implementation and gather data on current clinic characteristics (e.g. number of clinicians, involvement in national and local clinical initiatives, etc.). Sites will also submit their data on offering hydroxyurea 6 months after the study ends. These study procedures will be included in the study protocol. During weekly site calls, the Coordinating Team will develop a plan with each site to ensure this data continues to be collected in the post-award period using questions from the RE-AIM planning tool (http://re-aim.org/wp-content/uploads/2016/09/planningtool.pdf).

5. The virtual reality training simulation has not yet been adapted to SCD counseling for hydroxyurea and the extent of the development needs for the adaptation are unclear and could be substantial in terms of time and monetary resources. Please provide a detailed timeline for this process and a rationale for the use of virtual reality for this application.

At CCHMC, we used counseling in cases of influenza vaccine hesitancy as a proof of concept to assess the impact of virtual reality training on communication skills related to motivational interviewing. This

training resulted in a statistically significant decrease in rates of influenza vaccine refusal among providers that underwent this virtual reality curriculum when compared to those that did not.[13] Additionally, learners described the training as realistic, immersive, and fun. The communication skills taught during this curriculum included open-ended questioning, demonstrating empathy, and providing education without medical jargon. These communication skills are directly applicable to counseling in cases of hydroxyurea hesitancy. This virtual reality experience was created in approximately 12 weeks from start to final product. Since we will modify these scenarios for the purposes of this study, we anticipate that it would be completed in 8 weeks but have allotted an entire 12 weeks. Education that incorporates deliberate practice and standardization enhances clinician self-efficacy [14] because it improves clinical reasoning and communication skills.[15] We have included this information on page 8 of the application.

We have a summer research fellow who will be working on developing cases and mining qualitative data for phrases from existing parent interviews, provider interviews and clinic visit observations. This work will be completed using institutional funds. The research and VR team will be meeting in August to finalize the cases. Our STORM engagement group will be invited to these meetings, but if not convenient for engagement group members, we will review the cases and language during existing STORM engagement group meetings. Please see the Table below for a detailed timeline for the VR training component.

**Activity**	**Projected Completion Date**
**VR Milestone**	
Summer research fellow – develop cases and use language from existing interviews	July 2017
Finalize the cases and language for virtual reality training	August 2017
Pilot the virtual reality cases for content and messaging with key stakeholders including physicians, nurses, health educators and families and amend accordingly	September 2017
Create the shared decision making cases in the virtual reality platform, modified from the virtual reality created for the prior influenza study[13]	October 2017
Pilot the actual virtual reality scenarios to determine issues prior to clinical implementation	November 2017
Update the virtual reality scenarios based on pilot information to have the system ready for implementation	December 2018

6. **A few concerns were raised related to the sample size calculations. Please address.**

- **The application needs to provide justification for the sample size calculations. Did the calculations use the pilot data? Why is 0.6 a meaningful difference in this context? An ICC of 0.00001 is not very realistic and too low. For the ICC please provide preliminary data that supports that this estimation is correct or provide a power analysis table which should include a range of effect sizes, the variance of the effect, the level of significance, and minimum sample size given these expectations.**

  *Sample size calculation (**CI-2; CI-4; GM-5; CI-5**): We based our sample size calculation on minimal effect sizes basedon studies of the DCS (effect sizes range from 0.4 to 1.2),[1] our **primary outcome,** and a stepped wedge design (Hussey and Hughes approach)[16,17]. Specifically, power analyses were calculated using Optimal Design[18] power analysis software assuming: 1.) cluster (C = 3) level variance to be negligible due to within cluster balancing of sites based on size, 2.) site (J = 9) level variation at ICC = 0 following group-mean centering of all analysis variables, 3.) Ns between 207 (20% attrition) and 225 (ideal assuming ideal retention & proper missing data handling), 4.) effect sizes (δ) between 0.40-0.60, and 5.) the inclusion of control covariates (parent age, parent health literacy, participant gender, participant age, disease severity, & SES) will reduce response variable error variance by (R2 = 0.40) 40%. Results showed power will be $\geq$ 0.80 even if sample size (N = 180) and effect size (δ =.40) are at their minimal values if the treatment effect variation (σ^2_δ) is minimal ($\leq$0.037; see Table).*

J = 9 Sites	Effect Size (δ)		
R^2 = 0.40 for 6 covariates	0.40	0.50	0.60
N = 207 (n = 23)	0.037	0.116	0.213
N = 225 (n = 25)	0.045	0.124	0.219

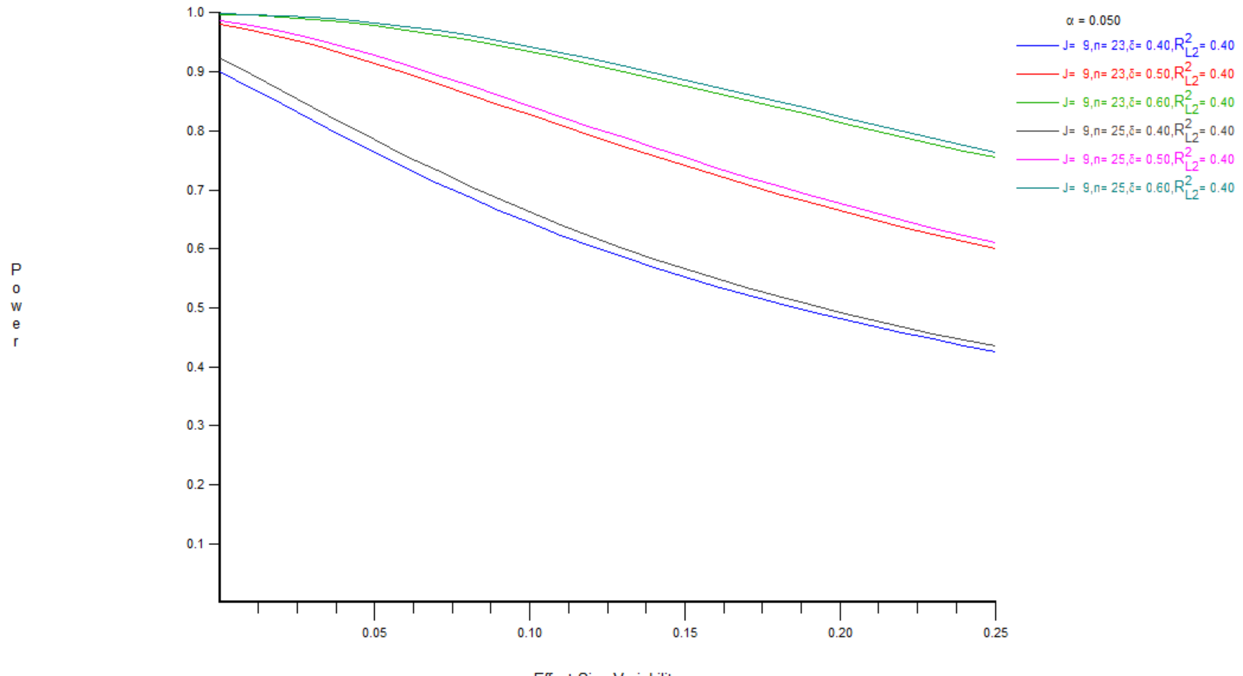

- **There is no discussion of or accounting for attrition.**

Participant attrition is an inherent problem in clinical trials with some reporting attrition rates up to 20%. We expect a 10% attrition rate across sites due to the low burden for participants in our study (surveys will be available on REDCap and study visits will occur during regularly scheduled clinic visits). Our eligible pool of 500 potential patients is sufficient for our targeted recruitment rate of 207 (225 to account for attrition) which represents a 41% recruitment rate. This is a reasonable goal based on previous multisite randomized controlled trials (RCTs) with children with SCD. Moreover, a systematic review of Sickle Cell Related RCTs registered on clinicaltrials.gov found that 47% of trials had an enrollment rate of ≥ 90%, 24% had a 60–90% enrollment rate with only 29% of RCTs enrolling <60%. For Phase 3 trials, 60% had enrollment rates ≥90%. We anticipate that our enrollment rate will match those of other trials (at least 60%).

We have proposed a 10% attrition rate because we will employ evidence-based strategies for optimizing retention and targeting non-respondents [19]. These include seeking out non-respondents to identify barriers, develop creative solutions and share those with the sites and teams during weekly study meetings. We will also schedule follow-up visits that coincide with routine clinic visits and schedule phone or video problem-solving Skype sessions at a convenient time for the families (e.g., evenings) – our Institution uses the HIPAA compliant Skype for Business program. We will collect multiple forms of contact information (e.g., cell phone numbers, emails, Facebook pages) from multiple contacts (e.g., family members, friends) to stay in close contact with families. Finally, we are using a graduated incentive system for study visits to reduce attrition.

7. **There were a few concerns related to the analytics for this application. Please address.**
    - **The only model considered is a linear mixed model, which is not suitable for categorical data such as hydroxyurea offered.**

*<u>Data Analysis for Aim 1 (**GM-3; CI-4; CI-5; MD-5**)</u>: The data will be analyzed based on the intent-to-treat principle. All patients will remain in the arm of the study to which they were randomized, regardless of whether or not they receive the assigned dissemination method. We will report our findings using the Consolidated Standards of Reporting Trials (CONSORT) statement extension to cluster randomized trials [20]. Characteristics of the clusters (e.g. size of population number of clinicians) and participants (e.g. health literacy, demographics) will be summarized using descriptive statistics. We will also assess fidelity during the H-SDM toolkit dissemination period and contamination during the usual care period by reviewing audio recordings of clinic visits.*

Data from all participants at all time-points will be aggregated within treatment group assignment and analyzed in two steps. First, acknowledging that J = 3 sites (1 "small", 1 "medium" and 1 "large" site) each will be assigned to C = 3 clusters in a manner balanced by site size, we will assume response variable variance across the C = 3 clusters to be negligible, and response variable variation across the J = 9 total sites will be eliminated (i.e., the ICC = 0) following group mean centering of all analysis variables at their respective site means[21] (alternative methods, such as 'Type = Complex' in Mplus, perform poorly if the number of nesting units, such as sites, is < 20). This will allow the effect of site-level clustering to be ignored and the effect of treatment group randomization to be assessed at the participant level without fear of inferential statistical test bias. Specifically, a multiple group comparison analysis will be performed in Mplus (Version 8) to assess significant response variable differences by group randomization. Further, missing data will be handled using multiple group multiple imputation (with M = 100 imputed datasets)[22] consistent with currently accepted methodological practice.[23]

*<u>Data Analysis (**GM-3; CI-4; CI-5**)</u>: The secondary outcomes of hydroxyurea knowledge and child reported health outcomes will be evaluated using similar methods to eliminate site level variance prior to data analysis. Specifically, we will analyze response variables (hydroxyurea offered, hydroxyurea uptake) as binary categorical and convert parameter estimates to odd ratios for interpretation. Further, forthe measures related to offering*

hydroxyurea and uptake, we will convert run chart data to control charts to determine if the process of offering hydroxyurea is under control (minimal variation in the data) and any special cause changes (i.e. factors that change the process significantly). Upper and lower control limits will be calculated as 3 sigma from the mean (e.g. standard Shewhart chart method)[24]. Any data point outside the control limits will be considered variation from a special cause. During the ***H-SDM toolkit*** *period, these data will be tracked on a monthly run chart (percent offered/percent eligible, and percent with active prescription/percent eligible). Run charts provide a graphic display of process performance over time to motivate and inform practice changes. Finally, additional healthcare utilization variables (number of hospitalizations, ill visits, and emergency room visits) will be analyzed as count variables and examined in exploratory analyses.*

- **It is not clear why and how certain sites are clustered together.**

We will randomly assign sites to the 3 clusters (3 sites per cluster). The randomization will guarantee that each cluster has a site considered large, medium and small (<270 patients = small; >270 – 500 = medium; >501 = large). This information is now included on page 17 and throughout the application.

- **The effect size characterization for the toolkit testing is incorrect for two of the three outcomes in the toolkit pilot testing.**

In our pilot study, we tested the H-SDM toolkit dissemination method with clinicians of parents newly facing the decision to initiate hydroxyurea locally and at a second Midwest SCA clinic (intervention group, n = 27). Parents completed the 16-item decisional conflict scale (DCS) to assess decisional uncertainty [25] and a 9-item survey to assess hydroxyurea knowledge specific to content of the decision aids (highest score 9). We evaluated acceptability using a 10-item survey used in previous studies [26]. Concurrently, parents from a

third pediatric SCA clinic in the West whose clinicians used usual care completed the DCS and hydroxyurea knowledge measures (control group, n = 20).

*We examined changes in DCS scores and hydroxyurea knowledge with repeated measures ANOVAs for the intervention group (pre and post). All parents using the decision aids reported that they were useful in decision-making (100%). Hydroxyurea knowledge increased and decisional conflict decreased pre to post using the decision aids (Table 3). We used an independent samples ANOVA to determine effect sizes (Cohen's d ≤0.2 = small; ≤0.5 = medium; ≤ 0.8 = large) [27] for hydroxyurea knowledge and DCS for parents whose clinicians did or did not use the H- SDM toolkit. Analyses revealed that parents whose clinicians used the H-SDM toolkit had lower scores on the DCS informed subscale (Table 3). Comparisons also revealed medium to large effect sizes for the DCS total scale (d =0.41) and DCS informed subscale (d=0.66). Given the DCS effect size of 0.41 and 0.66 for the informed subscale with such a small sample and the range of effect sizes for the DCS reported in the literature (*effect sizes range from 0.4 to 1.2)[1]*, we chose an effect size 0.60 to use in our sample size calculation.*

References

1. O'Connor A. User Manual—Decisional Conflict Scale. 1993 [updated 2010]. http://decisionaid.ohri.ca/docs/develop/User_Manuals/UM_Decisional_Conflict.pdf.
2. Melbourne E, Sinclair K, Durand M-A, Légaré F, Elwyn G. Developing a dyadic OPTION scale to measure perceptions of shared decision making. *Patient education and counseling.* 2010;78(2):177-183.
3. Melbourne E, Roberts S, Durand M-A, Newcombe R, Légaré F, Elwyn G. Dyadic OPTION: Measuring perceptions of shared decision-making in practice. *Patient education and counseling.* 2011;83(1):55-57.
4. Légaré F, Ratté S, Stacey D, et al. Interventions for improving the adoption of shared decision making by healthcare professionals. *The Cochrane Library.* 2010.
5. Cascardi M, Poythress NG, Hall A. Procedural justice in the context of civil commitment: An analogue study. *Behavioral sciences & the law.* 2000;18(6):731-740.
6. Walton A, Shook LM, Treadwell M, et al. Shared Decision Making for Hydroxyurea with Families of Patients with Sickle Cell Anemia. 10th Annual Sickle Cell Disease Research and Educational Symposium and 39th National Sickle Cell Disease Scientific Meeting; April 2016; Hollywood, Florida.
7. Panepinto JA, Torres S, Bendo CB, et al. PedsQL™ sickle cell disease module: feasibility, reliability, and validity. *Pediatric blood & cancer.* 2013;60(8):1338-1344.
8. Squires J, Bricker D, Potter L. Revision of a parent-completed developmental screening tool: Ages and Stages Questionnaires. *Journal of Pediatric Psychology.* 1997;22(3):313-328.
9. Weiss BD, Mays MZ, Martz W, et al. Quick assessment of literacy in primary care: the newest vital sign. *The Annals of Family Medicine.* 2005;3(6):514-522.
10. Osborn CY, Weiss BD, Davis TC, et al. Measuring adult literacy in health care: performance of the newest vital sign. *American Journal of Health Behavior.* 2007;31(1):S36-S46.
11. Elwyn G, Hutchings H, Edwards A, et al. The OPTION scale: measuring the extent that clinicians involve patients in decision-making tasks. *Health expectations.* 2005;8(1):34-42.
12. Brinkman WB, Majcher JH, Poling LM, et al. Shared decision-making to improve attention-deficit hyperactivity disorder care. *Patient education and counseling.* 2013;93(1):95-101.
13. Real FJ, DeBlasio D, Beck AF, et al. A Virtual Reality Curriculum for Pediatric Residents Decreases Rates of Influenza Vaccine Refusal. *Academic Pediatrics.* 2017.
14. Lopreiato JO, Sawyer T. Simulation-based medical education in pediatrics. *Academic pediatrics.* 2015;15(2):134-142.
15. Consorti F, Mancuso R, Nocioni M, Piccolo A. Efficacy of virtual patients in medical education: a meta-analysis of randomized studies. *Computers & Education.* 2012;59(3):1001-1008.
16. Hemming K, Haines T, Chilton P, Girling A, Lilford R. The stepped wedge cluster randomised trial: rationale, design, analysis, and reporting. *BMJ.* 2015;350:h391.

17. Hussey MA, Hughes JP. Design and analysis of stepped wedge cluster randomized trials. *Contemporary clinical trials.* 2007;28(2):182-191.
18. *Optimal design plus empirical evidence: Documentation for the "Optimal Design" software* [computer program]. 2011.
19. Flores G, Portillo A, Lin H, et al. A successful approach to minimizing attrition in racial/ethnic minority, low-income populations. *Contemporary Clinical Trials Communications.* 2017;5:168-174.
20. Campbell MK, Piaggio G, Elbourne DR, Altman DG. Consort 2010 statement: extension to cluster randomised trials. *Bmj.* 2012;345:e5661.
21. Enders CK, Tofighi D. Centering predictor variables in cross-sectional multilevel models: a new look at an old issue. *Psychol Methods.* 2007;12(2):121.
22. Graham JW, Olchowski AE, Gilreath TD. How many imputations are really needed? Some practical clarifications of multiple imputation theory. *Prevention Science.* 2007;8(3):206-213.
23. Enders CK, Gottschall AC. Multiple imputation strategies for multiple group structural equation models. *Structural Equation Modeling.* 2011;18(1):35-54.
24. Langley GJ, Moen R, Nolan KM, Nolan TW, Norman CL, Provost LP. *The improvement guide: a practical approach to enhancing organizational performance.* John Wiley & Sons; 2009.
25. O'Connor AM. Validation of a decisional conflict scale. *Medical decision making.* 1995;15(1):25-30.
26. O'Connor A, Cranney A. User Manual. *Ottawa, ON: Ottawa Hospital Research Institute.* 2002.
27. Cohen J. *Statistical power analysis for the behavioral sciences Lawrence Earlbaum Associates.* 1988.
